# Modern Hospital and Institutional Fittings.—I

**Published:** 1903-10-24

**Authors:** 


(*To be continued.*)

### MODERN HOSPITAL AND INSTITUTIONAL FITTINGS.—I.

### REFRIGERATION AND COLD STORAGE.

ALTHOUGH refrigeration has been known and used for many ages, it is only comparatively recently that it has been brought to such a pitch of perfection that it can economically be applied to industrial and allied purposes. Now, enormous quantities of ice can be manufactured more cheaply, more palatable and purer than the natural product; freshly killed meat can be frozen, transported and kept for an indefinite period, stores can be cooled, and in fact mechanical refrigeration is now commonly employed in most manufactories.

Chocolate manufacture, breweries, paraffin and candle works, photography, indiarubber works, tunnelling and laying foundations, all now employ refrigeration as an important element in their work, and without which they would be greatly handicapped.

One of the most ancient methods of producing artificial cooling is by taking advantage of the fact that a solid undergoing dissolution into a liquid abstracts heat from its surroundings. The well-known apparatus for icing creams, etc., are of this type, the frigorific agent being usually a mixture of pounded ice and sodium chloride. This method, though it has been used extensively for ice-making and cooling, is somewhat expensive and cannot compare with the strictly mechanical methods we shall now speak of.

The mechanical refrigerating machines may be divided into two classes:—(1) Those using air, and (2) those using some chemical agent, having a low boiling point, which produces cold by alternate condensation and vaporisation.

1. The cold-air system:—Machines working on this system operate on one of the simplest principles of physics, viz., that the compression of air generates heat and its subsequent expansion cold. The process is as follows:—Atmospheric air is compressed till the pressure is about 50 lbs. to the square inch; the air is thus raised in temperature, and the heat is removed by passing the air through a "cooler" which is surrounded by circulating water. Thence the air passes to a "dryer," where moisture is deposited, and afterwards through a throttle valve to the "expansor", where it is suddenly allowed to expand (and in doing so helps to drive machinery), is reduced in temperature to below freezing point, and goes away through air trunks to the cold room.

The advantages of cold air machines are:—First, that no chemicals of any kind are required, consequently their employment is not attended by dangers of possible explosions or loss of life through the escape of noxious gases. Very low temperatures can be rapidly obtained by their use. Their construction is simple and their application easy. Against the system it must be said that it is not economical, for the co-efficient of performance is about three-quarters of a unit, whereas with a vapour compression machine it is four to five units. Nevertheless, modern improvements having overcome many of its previous drawbacks, the cold air system is somewhat extensively employed in marine installations, and to a less extent for refrigerating cold stores for the preservation of provisions of a perishable nature.

2. Systems involving the use of some chemical agent, having a low boiling point and producing cold by alternate condensation and evaporation. Separated into two divisions:—(*a*) The absorption machine and (*b*) the compression machine.

(*a*) *The Absorption Process.*—This is more a chemical or physical process than a mechanical one. It has for its basis the fact that water has a great capacity for absorbing many vapours which have a low boiling point, and which easily separate again by heating the combined liquid. Ammonia and sulphurous acid are the vapours which have most commonly been employed. In the case of the former, the working is as follows:—The ammonia and water contained in a generator are heated by means of a steam coil, when ammonia gas is driven off under pressure and is passed through an "analyser," where it is robbed of much of the watery vapour mixed with it; thence it is passed to a "rectifier," where all remaining traces of moisture are removed and where it liquefies. It now passes through a throttle valve into the "expansor," where the pressure being relieved it re-evaporates, and in doing so absorbs heat from brine circulating around it, which brine is pumped to the rooms required to be cooled. Brine is employed for this purpose because it is not liable to freeze. The ammonia vapour is re-absorbed by water and is returned to the generator, when the cycle again begins. Though this process can show a greater co-efficient of performance than the cold air system, yet it has less than the system next described.

(*b*) *The Compression Process.*—Here, the cycle of operation is as follows. First the refrigerating agent, in gaseous form, is compressed by machinery till it becomes liquefied, and the heat thus formed is carried off by cooling water, which surrounds the pipes containing the compressed gas. Next the liquefied gas is admitted to expansor pipes where it is suddenly relieved of pressure, and in suddenly expanding it takes up heat from its surroundings, firstly the pipes it is in, and secondly any substance around the pipes, such as brine. This cooled brine, as in the absorption process, is continually pumped to the storage chambers where the atmosphere can thus be cooled to a very low temperature.

The outlines of the principal systems of mechanical refrigeration having been described, it is now possible to compare their relative values and to understand what determines the choice of a refrigerating agent, the selection of which, as will be seen, depends to a large extent on the purpose for which refrigeration is required and on the adaptability of the necessary machinery to its surroundings.

Both the cold-air and absorption processes being less efficient than the compression system using a chemical agent, this latter is now almost entirely used. The main point to next consider is what this agent shall be.

Though many agents in the past have been used, viz., sulphurous acid, carbonic acid, ether, ammonia, methyl chloride, etc, nearly all the modern machines now employ either ammonia or carbonic acid gas. Where space is of no object the greatest efficiency, especially for ice-making, is obtained from ammonia gas, as though a large volume of the gas has to be compressed to produce a certain refrigerating effect, yet less pressure has to be exerted to bring about liquefaction, and these two factors practically govern the design of the machine. In the case of carbonic acid gas we have the exact reverse, viz., only a small volume of gas requiring compression to produce the same refrigerating effect, but a much larger pressure has to be exerted to liquefy it. This high pressure is only a mechanical effect, and can easily and readily be dealt with, while the small size of the compressor required is such an advantage where the space for machinery is limited that we find that carbonic acid machines are mostly in requisition in modern refrigerating installations. Other points in favour of the use of carbonic acid gas are its non-inflammability and non-explosive nature, its non-corrosive action on copper —which latter fact is of especial importance in marine installations. Ammonia gas, though of great value as a refrigerating agent, is explosive (when mixed with twice its volume of air), is most irritating if a leakage occur and has a corrosive action on copper. Sulphurous acid gas is still used to some extent, and ether, though a most expensive and disadvantageous method, has been used to some extent for military purposes, as it was in the late Boer war, on account of its portability.

